# Mechanism of Electroacupuncture Analgesia on Nicotine Withdrawal-Induced Hyperalgesia in a Rat Model

**DOI:** 10.1155/2022/7975803

**Published:** 2022-08-29

**Authors:** Jeimy Alfonso-Rodriguez, Shuju Wang, Xiaoling Zeng, Keith A. Candiotti, Yanping Zhang

**Affiliations:** ^1^Department of Anesthesiology, Perioperative Medicine and Pain Management, University of Miami Miller School of Medicine, Miami, USA; ^2^Hubei University of Chinese Medicine, Wuhan, Hubei, China

## Abstract

**Purpose:**

This study aimed to investigate the analgesic effect and mechanism of electroacupuncture (EA) in nicotine withdrawal-induced hyperalgesia rats.

**Methods:**

Behavioral testing was conducted twice a week for 7 weeks during nicotine administration using von Frey filaments. Electroacupuncture at the bilateral “Zusanli” and “Taichong” points was applied daily for 3 days during nicotine withdrawal. Western blot analysis and immunohistology were used to determine expression levels of pain-related factors in the spinal cord and midbrain periaqueductal gray (PAG).

**Results:**

Behavioral tests showed that electroacupuncture had a significant analgesic effect on nicotine withdrawal-induced hyperalgesic rats. Western blot results demonstrated that, in hyperalgesic rats, the expressions of nicotinic acetylcholine receptors (subunits: nAChR *α*7, *α*4, or *β*2) decreased in the spinal cord, nAChR *α*7, and *β*2 decreased in PAG. The proinflammatory factor cyclooxygenase 2 (COX2) and the activated microglia (ionized calcium-binding adaptor molecule 1, Iba1 positive cells) increased in the spinal cord and PAG compared to controls. After electroacupuncture treatment, nAChR *α*7 and nAChR *β*2 expressions increased significantly, and COX2 and Iba1 expressions decreased in the spinal cord. Compared with the nonelectroacupuncture nicotine withdrawal group, electroacupuncture stimulation increased the expression of nAChR *α*7 and nAChR *α*4 in the PAG of rats with electroacupuncture. Immunohistochemical results confirmed that electroacupuncture reversed nicotine withdrawal-induced changes in nAChR *α*7 positive neurons and Iba1-positive microglia in the dorsal horn of the spinal cord.

**Conclusion:**

Electroacupuncture treatment has an analgesic effect on nicotine withdrawal-induced pain in nicotine-dependent rats. The mechanism of analgesia of the electroacupuncture treatment relates to the increased expression of nAChR *α*7 and nAChR *β*2 proteins in the spinal cord, nAChR *α*7 in the PAG, and decreased expression of Iba1 and COX2 protein in the spinal cord.

## 1. Introduction

Nicotine dependence and chronic pain have been connected in research for several decades due to their economic burden and impact on societal health. Chronic pain and tobacco use are comorbid conditions that exist in a positive feedback loop where more pain results in a worsening addiction and vice versa [1]. Tobacco smoking has been reported to be a risk factor for the development of chronic pain. Studies have shown an increased frequency of chronic pain conditions among cigarette smokers, demonstrating a greater degree of pain and more painful locations compared to nonsmokers. Furthermore, in a cohort study, active smoking was the greatest predictor of future chronic back pain [2]. Conversely, pain can act as a motivator for smoking. Literature has shown that individuals with a lifetime history of chronic pain are significantly more likely to become smokers [3]. The prevalence of tobacco use in people with chronic pain is twice that of people without [1].

Chronic pain may be exacerbated during the early stages of nicotine withdrawal. Smokers are likely to experience hyperalgesia (regardless of long-term or short-term nicotine use) during the early stages of nicotine withdrawal, influencing both central and peripheral pain pathways [4]. Additionally, nicotine withdrawal increases the risk of opioid dependence in the postoperative period. During nicotine withdrawal, postoperative pain is more severe in smokers compared to nonsmokers, and smokers require more pain medication by comparison. In a study following patients requiring coronary artery bypass grafting, smokers required an overall 33% higher dose of opioids compared to nonsmokers [5].

Acupuncture is a form of treatment and prevention for many diseases in Traditional Chinese Medicine. The technique is straightforward and strategic points on the body are stimulated with thin needles inserted into the skin. The practice of acupuncture originated in China and can be traced back centuries ago and is used worldwide to treat pain. The theory behind acupuncture is based on the traditional belief that energy (Qi) flows within the body and can be channeled to create balance and improve health. There are 12 main channels, called meridians, and each point on the meridian represents a specific major organ [6]. Electroacupuncture is a type of acupuncture where small electric currents are passed through special needles. This technique is often used in pain treatment. Electroacupuncture blocks pain by activating a variety of bioactive chemicals through peripheral, spinal, and supraspinal mechanisms. Clinical and preclinical studies have shown that acupuncture can produce an analgesic effect in various pain disorders [7].

The purpose of this study was to observe the effect of electroacupuncture at “Zusanli” (stomach meridian) and “Taichong” (liver meridian) points on pain perception in a nicotine withdrawal-induced hyperalgesia rat model. In this study, we investigated the effect of electroacupuncture stimulation on the expression of pain-related molecules (glycine receptor alpha3 (GLRA3), cyclooxygenase 2 (COX2), microglia marker protein ionized calcium-binding linker 1 (Iba1), acetylcholine receptor *α*7 (nAChR *α*7), nAChR *α*4, and nAChR *β*2) to reveal the analgesic mechanisms of electroacupuncture.

## 2. Materials and Methods

### 2.1. Animals and Husbandry

The animal study protocols were approved by the University of Miami's Institutional Animal Care and Use Committee (IACUC). All efforts were made to minimize animal suffering and to reduce the number of animals used. Male Sprague-Dawley (SD) rats were purchased at 5 weeks old with a weight range of 130–150 g from Charles River Laboratories International, Inc. (USA). The rats were kept in pairs in a plastic cage lined with soft bedding and open access to food and water. The cage was maintained at 22 ± 2°C with 50–60% relative humidity, and the rats were exposed to a 12-hour light and 12-hour dark cycle. The rats were kept in cages for a one-week acclimation period before the experiment began. The rats were randomly divided into two groups, an age-matched control group (CTR, *n* = 6) and a nicotine-exposed group (NIC, *n* = 12). After the models of nicotine hyperalgesia were established, the nicotine-exposed rats were then randomly divided into a nicotine withdrawal group (NIC + *W*, *n* = 6) and a nicotine withdrawal/electroacupuncture (EA) treatment group (NIC + *W* + EA, *n* = 6).

### 2.2. Nicotine Administration

Generally, smokers start experimenting with nicotine during their teenage years. Their nicotine intake gradually increases as they grow older, and their addiction worsens [8]. This experiment is meant to replicate this process. SD rats begin puberty at around 6 weeks old; therefore, nicotine administration was started in this period. The method of nicotine administration is consistent with our previous method [4, 9]. In short, nicotine (Sigma, St. Louis, MO, USA) was dissolved in drinking water, and the dose was adjusted through strict inspection of the daily water intake. Nicotine was dosed at 1 mg/kg/day in the first week, 1.2 mg/kg/day in the second week, 1.5 mg/kg/day in the third week, and 2 mg/kg/day after the fourth week (4–7 weeks). This amount of nicotine is equivalent to the amount consumed by a long-term smoker [10]. In the eighth week, nicotine was withdrawn. The first electroacupuncture treatment was given 24 hours after nicotine withdrawal and was then given at the same time every day for the next two days.

### 2.3. Behavioral Testing

In order to evaluate changes in mechanical sensitivity after nicotine administration and electroacupuncture, von Frey filaments (North Coast Medical, Inc., USA) were applied to the hind paws of rats to measure mechanical withdrawal thresholds. Tests were performed twice a week during the 7 weeks of nicotine exposure and daily during the 3 days of electroacupuncture treatment. Behavioral baseline values were measured twice in the week before nicotine exposure began. Rats were placed on a clear plastic-covered wire-mesh platform and were given 20 minutes for habituation. Testing began with either the left or the right paw, and the filament was applied for 5 seconds each. A positive reaction (paw withdrawal, flinching, or licking) was followed by a thinner filament application. A negative reaction was followed by a thicker filament. The responses were measured 5 times, and then the mean value was calculated. The hind paw mechanical withdrawal threshold was measured in grams.

### 2.4. Electroacupuncture (EA) Treatment

**Figure 1:**
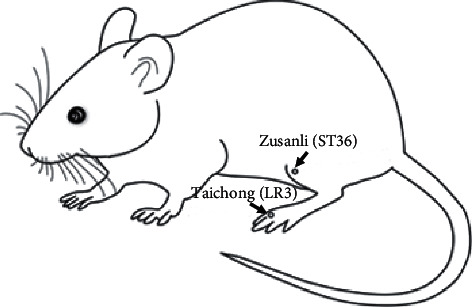
The location of the acupuncture points ST36 and LR3.

All of the nicotine withdrawal rats in the NIC + *W* + EA group began to receive electroacupuncture at the bilateral “Zusanli” (ST36) and “Taichong” (LR3) points 24 hours after their nicotine withdrawal period. As shown in Figure 1, the “Zusanli” (ST36) acupoint is 5 mm below the fibular head and posterolateral to the knee joint. The “Taichong” (LR3) acupoint is in between the metatarsals of the foot on the dorsal surface. A 0.25 mm × 13 mm stainless steel needle (Huatuo Medical Instruments Co., Ltd., Suzhou, China) was used. The needles were inserted at the “Zusanli” (ST36) acupoint at an angle of 90° and a depth of 7 mm. The second set of needles were inserted at the “Taichong” (LR3) acupoint at an angle of 45° and a depth of 4 mm. A G6805-2 electroacupuncture apparatus (Shanghai Medical Instruments Co., Ltd., Shanghai, China) was used with a frequency of 2 Hz and an intensity of 1 mA. The treatments were administered 20 minutes daily for 3 days. The electroacupuncture administered to the NIC + *W* + EA group was applied at the same time and by the same researcher every day. The control and NIC + *W* groups experienced grasping and fixation similar to the NIC + *W* + EA group but without electroacupuncture treatment. Behavioral baseline values were measured once before the first electroacupuncture treatment. Five hours after each electroacupuncture treatment, mechanical sensory behavioral tests were performed in all three groups of rats.

### 2.5. Immunohistochemistry and Image Preparation

Spinal cords from the rats (3 groups, *n* = 4) were harvested after perfusion with 4% paraformaldehyde in 0.1 M phosphate buffer (PBS, pH 7.4), dehydrated with 30% sucrose, placed in tissue-embedding gel, and cut into 20-*μ*m spinal cord slices by cryosection. Sections were incubated with primary antibodies (rat anti-nAChR *α*7 and rabbit anti-Iba1; Millipore, USA) overnight and, after 3 washes (PBS), were incubated with fluorescent-conjugated secondary antibodies (anti-rat-rhodamine and anti-rabbit-FITC; Jackson ImmunoResearch Lab, USA) for one hour. Double-stained dorsal horn sections of the spinal cord were imaged under a Leica fluorescence microscope. nAChR *α*7-positive neurons and Iba1-stained microglia were counted in a fixed-size area (1600 × 1200 pixels) in the dorsal horn of the spinal cord.

### 2.6. Western Blot Analysis

Lumbar spinal cord and periaqueductal gray (PAG) tissue samples were dissected after an overdose of isoflurane anesthesia and then promptly frozen and stored at −80°C.

Samples were homogenized in RIPA lysis buffer (composed of 1 protease inhibitor and 2 phosphate inhibitors). The homogenate was centrifuged at 4°C at 18,000*g* for 20 minutes, and the supernatants were extracted and transferred into new tubes. Protein concentrations were measured and diluted with water, Laemmli loading buffer (Bio-Rad, USA), and beta-mercaptoethanol (Sigma-Aldrich, USA). The mixtures were denatured at 98°C for 5 minutes. Proteins were separated using 10% tris-glycine SDS-PAGE during gel electrophoresis and transferred to polyvinylidene fluoride (PVDF) membranes during electrotransfer. Membranes were blocked using Western Blocking Reagent (Sigma-Aldrich, USA) for 1 hour and incubated in a primary antibody at 4°C overnight. The primary antibodies used were mouse anti-GAPDH (1 : 2,000, Sigma, USA) as the loading control and mouse anti-COX2 (1 : 100, Santa Cruz Biotechnology, USA), rat anti-nAChR *α*7 (1 : 200, Millipore, USA), rabbit anti-nAChR *α*4 (1 : 500, NovusBio, USA), rabbit anti-nAChR *β*2 (1 : 350, Aviva Systems Biology, USA), rabbit anti-Iba1 (1 : 2000, Bio-Rad, USA), and rabbit anti-GLRA3 (1 : 400, Aviva Systems Biology, USA). Membranes were washed 3 times with phosphate-buffered saline solution with 0.05% Tween-20 (PBST) for 10 minutes before secondary antibody incubation at room temperature for 2 hours. The horseradish peroxidase (HRP) conjugated secondary antibodies used were anti-mouse (1 : 3,000, Santa Cruz Biotechnology, USA), anti-rabbit (1 : 4,000, Santa Cruz Biotechnology, USA), and anti-rat (1 : 2,000, Santa Cruz Biotechnology, USA). Membranes were rewashed 3 times with PBST for 10 minutes before being incubated with a chemiluminescence solution composed of Luminol and Peroxide (Thermo Scientific, USA). The membranes were quantified using an Image Lab system (Bio-Rad, USA), and target bands were normalized using internal controls.

### 2.7. Statistical Analysis

Analysis of western blots and immunohistological images were blinded to reduce bias. Statistical analyses were performed using GraphPad Prism version 6.0 software (GraphPad Software, San Diego, CA, USA). Behavioral testing data were presented as mean ± standard error of mean (SEM). They were analyzed via a two-way analysis of variance followed by Bonferroni post hoc tests to compare the differences over time. Western blot data were presented as relative values to control and analyzed using Student's *t*-test. Group comparisons of immunohistological images were made for the number of nAChR*α*7-positive neurons and Iba1-stained microglia in fixed-size regions of the spinal dorsal horn. One-way analysis of variance was applied. *p*-values of less than 0.05 were considered statistically significant and are presented along with the data.

## 3. Results

### 3.1. Mechanical Sensory Effects of Electroacupuncture in Nicotine Withdrawal-Induced Hyperalgesic Rats

**Figure 2:**
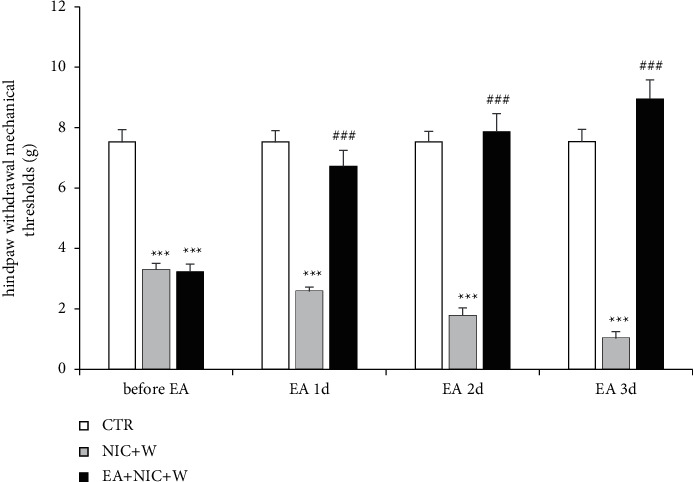
Mechanical sensory effects of electroacupuncture in nicotine withdrawal-induced hyperalgesic rats. Behavior tests were analyzed using two-way analysis of variance followed by Bonferroni post hoc tests to compare the differences over time (CTR: *n* = 6, NIC + *W*: *n* = 6, NIC + *W* + EA: *n* = 6). (Compared to CTR group: ^*∗∗∗*^*p* < 0.001, Compared to NIC + *W* group: ^###^*p* < 0.001). CTR—control group, NIC + *W*—nicotine withdrawal group, NIC + *W* + EA—nicotine withdrawal with electroacupuncture group, EA—electroacupuncture.

Behavioral test results in the three groups of rats were evaluated during the 8^th^ week: age-matched control rats (CTR, *n* = 6), nicotine withdrawal rats (NIC + *W*, *n* = 6), and the nicotine withdrawal/electroacupuncture treatment group (NIC + *W* + EA, *n* = 6) were compared. A von Frey filaments test was performed once every day, 5 hours after each electroacupuncture treatment.  Figure 2 shows the differences between the 3 groups. The NIC + *W* group consistently demonstrated lower sensory thresholds than the CTR group (*p* < 0.001). After electroacupuncture treatment, the mechanical thresholds increased and were significantly higher than those in the NIC + *W* group without electroacupuncture (*p* < 0.001), indicating that electroacupuncture had an analgesic effect.

### 3.2. Effects of Electroacupuncture on nAChR *α*7, nAChR *α*4, nAChR *β*2, GLRA3, COX2, and Iba1 Protein Expressions in the Spinal Cords of Withdrawal-Induced Hyperalgesic Rats

**Figure 3:**
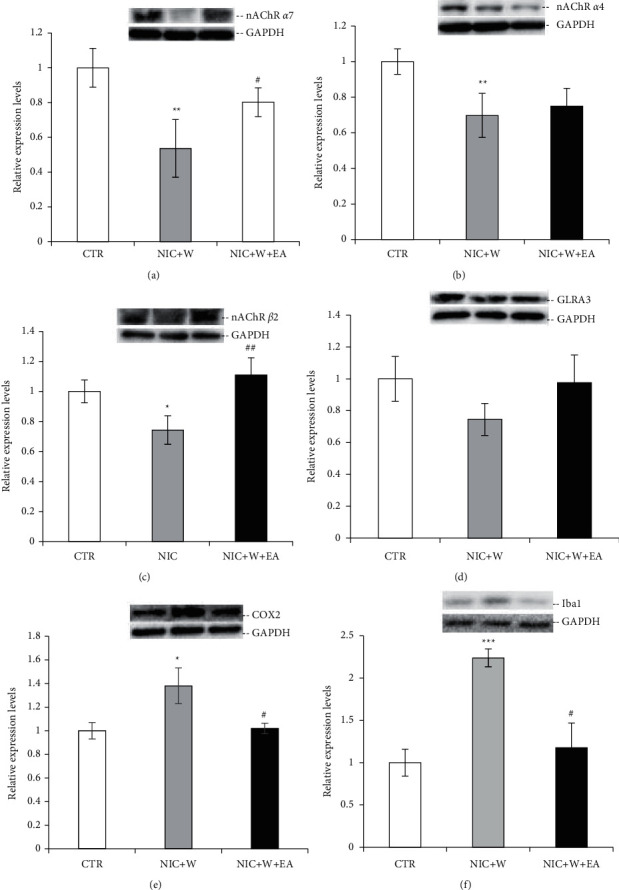
Electroacupuncture effects on nAChR *α*7, nAChR*α*4, nAChR *β*2, GLRA3, COX2, and Iba1 protein expressions in the Spinal Cords. Western blot analysis of nAChR *α*7 (a), nAChR *α*4 (b), nAChR *β*2 (c), GLRA3 (d), COX2 (e), and Iba1 (f) in the spinal cord of rats in the three groups (CTR: *n* = 6, NIC + *W*: *n* = 6, NIC + *W* + EA: *n* = 6). Statistical tests were analyzed using Student's *t*-test. (^*∗*^*p* < 0.05, ^*∗∗*^*p* < 0.01, ^*∗∗∗*^*p* < 0.001 vs. the CTR group; ^#^*p* < 0.05, ^##^*p* < 0.01, vs. the NIC + *W* group). CTR—control group, NIC + *W*—nicotine withdrawal group, NIC + *W* + EA—nicotine withdrawal with electroacupuncture group.

nAChR *α*7, nAChR *α*4, nAChR *β*2, GLRA3, COX2, and Iba1 protein expression levels in the spinal cord of rats (CTR: *n* = 6, NIC + *W*: *n* = 6, NIC + *W* + EA: *n* = 6) were measured after 3 days of electroacupuncture treatment. In the NIC + *W* group, nAChR *α*7 (Figure 3(a)), nAChR *α*4 (Figure 3(b)), and nAChR *β*2 (Figure 3(c)) showed significantly lower protein expression levels than the respective CTR group, but COX2 (Figure 3(e)) and Iba1 (Figure 3(f)) showed significantly higher protein expression levels than the CTR group, indicating that nicotine withdrawal is partly responsible for the changes in pain-related protein expression levels. After electroacupuncture, in the group of NIC + *W* + EA, protein expression levels of nAChR *α*7 (Figure 3(a)) and nAChR *β*2 (Figure 3(c)) were significantly higher than the NIC + *W* group that did not receive electroacupuncture. The protein expression levels of COX2 (Figure 3(e)) and Iba1 (Figure 3(f)) were significantly lower in the NIC + *W* + EA group compared to the NIC + *W* group that did not receive electroacupuncture. There was no significant difference in the protein expression levels of nAChR *α*4 (Figure 3(b)). Inhibitory glycine receptors (GLRA3) also showed a decreased expression in NIC + *W* rats, and electroacupuncture treatment caused a numerical increase, but it did not reach statistical significance (Figure 3(d)). The results demonstrate that nAChR *α*7 and nAChR *β*2 protein expression levels were significantly increased and that COX2 and Iba1 protein expression levels were significantly decreased after electroacupuncture treatment in the spinal cord of nicotine withdrawal-induced hyperalgesic rats. This indicates that the analgesic effects of electroacupuncture on nicotine withdrawal-induced pain may occur by regulating the expression of these pain-related molecules in the spinal cord.

### 3.3. Electroacupuncture Effects on nAChR *α*7, nAChR *α*4, nAChR *β*2, GLRA3, COX2, and Iba1 Protein Expressions in the PAG of Withdrawal-Induced Hyperalgesic Rats

**Figure 4:**
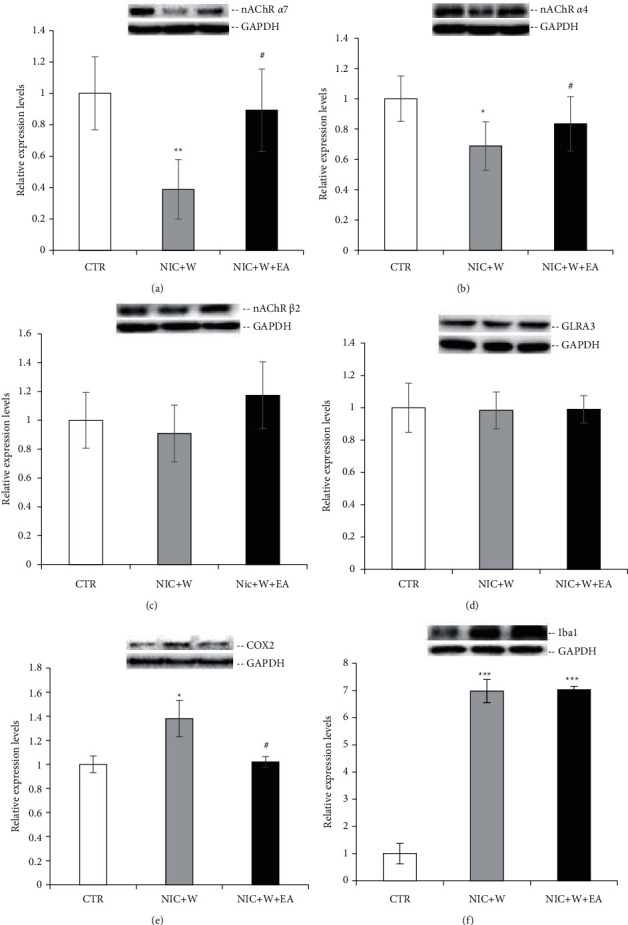
Electroacupuncture effects on nAChR *α*7, nAChR *α*4, nAChR *β*2, GLRA3, COX2, and Iba1 protein expressions in the PAG. Western blot analysis of nAChR *α*7 (a), nAChR *α*4 (b), nAChR *β*2 (c), GLRA3 (d), COX2 (e), and Iba1 (f) in the PAG of rats in the three groups (CTR: *n* = 6, NIC + *W*: *n* = 6, NIC + *W* + EA: *n* = 6). Statistical tests were analyzed using Student's *t*-test. (^*∗*^*p* < 0.05, ^*∗∗∗*^*p* < 0.001 vs. the CTR group; ^#^*p* < 0.05 vs. the NIC + *W* group). CTR—control group, NIC + *W*—nicotine withdrawal group, NIC + *W* + EA—nicotine withdrawal with electroacupuncture group.

nAChR *α*7, nAChR *α*4, nAChR *β*2, GLRA3, COX2, and Iba1 protein expression levels in the PAG of rats (CTR: *n* = 6, NIC + *W*: *n* = 6, NIC + *W* + EA: *n* = 6) were measured after 3 days of electroacupuncture treatment. The NIC + *W* demonstrated lower expression levels of nAChR *α*7 (Figure 4(a)) and nAChR *α*4 (Figure 4(b)) and a higher expression level of COX2 (Figure 4(e)) and Iba1 (Figure 4(f)) compared to the CTR group, suggesting that nicotine withdrawal influences pain perception through affecting the expression of nAChR *α*7, nAChR *α*4, COX2, and Iba1. There was no significant difference in the protein expression levels of nAChR *β*2 (Figure 4(c)) or GLRA3 (Figure 4(d)) in the NIC + *W* group compared to the CTR group. After electroacupuncture, the protein expression levels of nAChR *α*7 (Figure 4(a)) and nAChR *α*4 (Figure 4(b)) were significantly higher than in the NIC + *W* group that did not receive electroacupuncture, and protein expression levels of Iba1in the NIC + *W* + EA group (Figure 4(f)) were still significantly higher than in the CTR group. Meanwhile, there was no significant difference in the protein expression levels of nAChR *β*2 (Figure 4(c)), GLRA3 (Figure 4(d)), or COX2 (Figure 4(e)) and Iba1 (Figure 4(f)) in the NIC + *W* group compared to the NIC + *W* + EA group. The results suggest that nAChR *α*7 and nAChR *α*4 expression levels were significantly increased in the PAG after electroacupuncture treatment of withdrawal-induced hyperalgesic rats, indicating that the levels of nAChR *α*7 and nAChR *α*4 in the PAG are closely related to the levels of hyperalgesia.

### 3.4. Differences in nAChR *α*7-Positive Neurons and Iba1-Stained Microglia in Each Group

**Figure 5:**
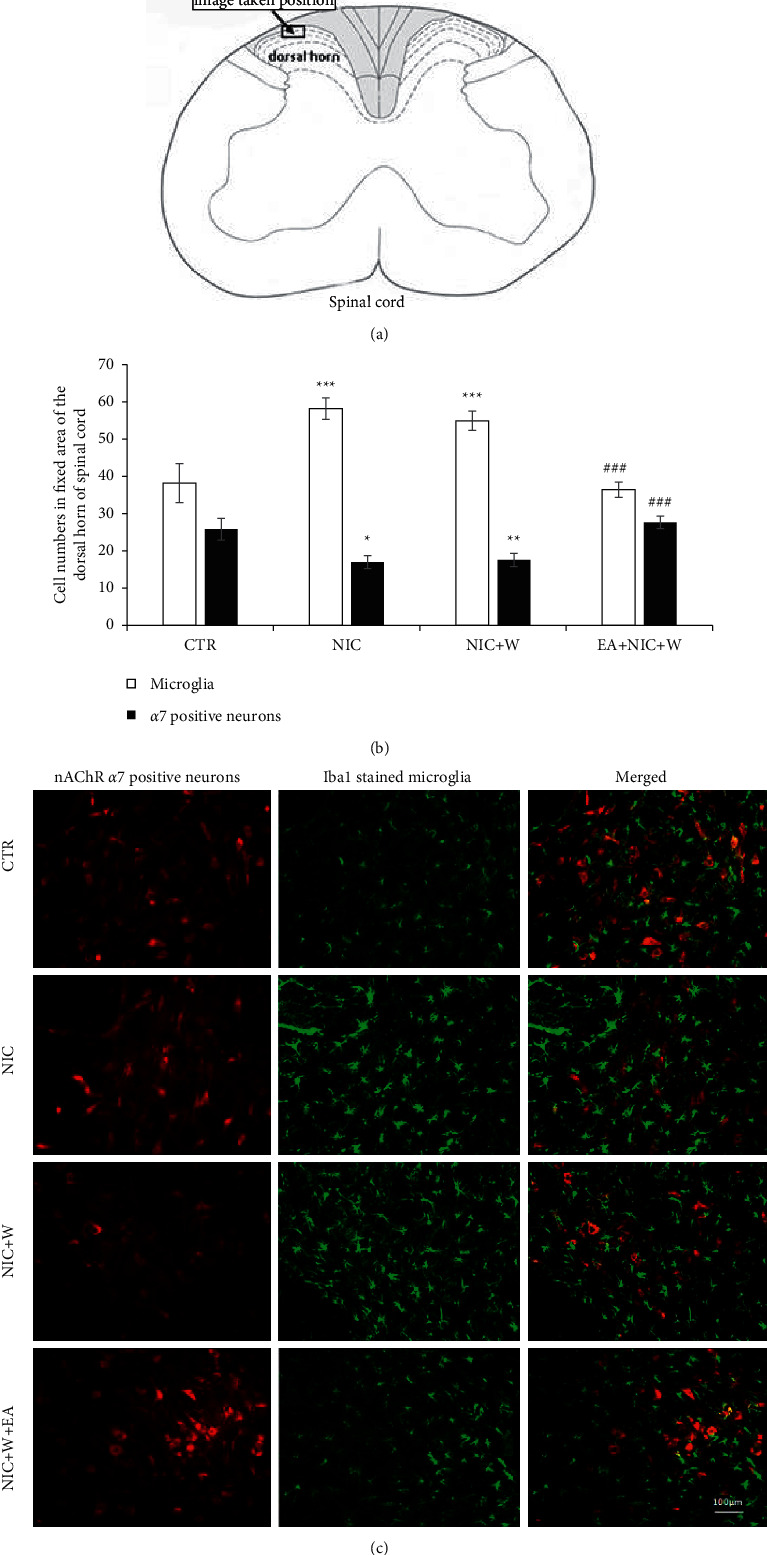
Spinal dorsal horn nAChR *α*7 neurons and Iba1-stained microglia. (a) Shows the location of the image taken and the area of dorsal horn cell counts. (b) Is a histogram of the number of nAChR*α*7 neurons and Iba1-stained microglia in each group of rats in the same region. ^*∗*^*p* < 0.05, ^*∗∗*^*p* < 0.01, ^*∗∗∗*^*p* < 0.001 vs. control, ^###^*p* < 0.001 vs. NIC + *W* group, in the same type of cells. (c) Shows double immunohistochemical staining for anti-nAChR*α*7 and anti-Iba1. The scale bar in the image is 100 *μ*m. CTR—control group, NIC + *W*—nicotine withdrawal group, NIC + *W* + EA—nicotine withdrawal with electroacupuncture group.

Figure 5(a) shows the area of spinal cord dorsal horn cell counts.  Figure 5(b) is a histogram of the number of nAChR *α*7 neurons and Iba1-stained microglia in each group and in the same area. As can be seen, electroacupuncture reversed the nicotine withdrawal-induced significant reduction in nAChR *α*7 neurons and reduced the increase in microglia induced by nicotine withdrawal (*p* < 0.05, Student's *t*-test). These results are consistent with the above results at the protein level.  Figure 5(c) is an image of immunofluorescence staining of the spinal dorsal horn for each group.

## 4. Discussion

This study used hyperalgesia induced by nicotine withdrawal in nicotine-dependent rats as a model to study the effects of electroacupuncture on pain. Behavioral results showed that nicotine withdrawal-induced hyperalgesia was present and subsequent electroacupuncture treatment had a positive analgesic effect. To study the mechanisms of electroacupuncture analgesia, the expression of pain-related molecules was examined. The results demonstrated that, in the spinal cord and PAG, nicotine withdrawal resulted in decreased protein expression of nicotinic acetylcholine receptors (nAChR *α*7, nAChR *α*4, nAChR *β*2) but significantly increased proinflammatory factors COX2 and Iba1 compared with controls. After electroacupuncture in nicotine withdrawal rats, the protein expressions of nAChR *α*7, nAChR *β*2, GLR, COX2, and Iba1 all returned to the levels of the control group for the spinal cord. This shows that electroacupuncture increased the expression of antipain molecules but suppressed the expression of proinflammatory factors in the spinal cord. Similar results were also presented in PAG, but the expression of Iba1 was still high; electroacupuncture treatment did not reduce the high expression of inflammatory factor Iba1 in PAG. The immunohistochemical results showed that the number of nAChR *α*7 neurons were significantly decreased, while Iba1-positive microglia were significantly increased in the spinal cord of nicotine-withdrawal rats. Electroacupuncture stimulation increased the number of nAChR *α*7 neurons and reduced Iba1 microglia numbers, thereby reducing the inflammatory response in the spinal cord. The results of this study suggest that modulation at the spinal cord level plays an important role in nicotine withdrawal-induced hyperalgesia, with nAChR *α*7 neurons and microglia being key factors.

Nicotine is released during cigarette smoking, and repeated exposure to nicotine can lead to dependence. Nicotine dependence and chronic pain are comorbid conditions. People with chronic pain have a higher smoking rate than those without chronic pain, and about 60% of tobacco users meet the criteria for chronic pain [11]. A study evaluating the postoperative pain levels of smokers showed that they exhibited more severe pain before and after surgery than nonsmokers and required higher doses of opioids after surgery [12]. Opioid use for pain management has led many patients to develop opioid addiction and dependence. Cyclooxygenase 2 (COX2) is the main source of prostaglandin (PG) induced by inflammation. However, it has been reported that COX2 is upregulated in the spinal cord after nerve injury [13, 14]; the effects of smoking and nicotine addiction on COX2 expression in the spinal cord and PAG have not been previously reported. Iba1 is a marker protein of microglial activation [15]. This study showed that long-term nicotine use, followed by nicotine withdrawal, increased COX2 expression and activated microglia in the spinal cord and PAG. These findings indicate that high levels of neuroinflammation may contribute to the hyperalgesia noted in smokers. Further, it appears that electroacupuncture treatment can reduce neuroinflammation in the spinal cord and PAG.

Acupuncture has been used for thousands of years in China and is now accepted as a pain relief method in many countries. The practice of acupuncture includes inserting needles into the skin at specific acupoints. In a meta-analysis, acupuncture had a statistically significant benefit in the treatment of chronic pain [16]. Acupuncture can also serve as a partial replacement for opioids to treat postoperative pain. Acupuncture has been deemed as effective as morphine and a much safer method of pain relief than relying on opioids [17]. Electroacupuncture is a type of acupuncture that adds electrical current to the needles. The electrical charge is meant to substitute the need for manual manipulation of the needles. Electroacupuncture has been used extensively as a holistic treatment due to its promising results in regulating inflammatory responses [18]. Studies have shown that the mechanism of the analgesic effect of acupuncture is mediated by central neurotransmitters. Among them, endogenous opioid peptide (EOP) plays an important role in acupuncture analgesia [19]. In addition to endogenous opioids, researchers have found that electroacupuncture activated the descending serotonergic pathways to inhibit the release of substance *p*, thereby producing significant analgesia [20]. Therefore, the serotonergic descending inhibitory pathway and endogenous opioids appear to act synergistically, resulting in acupuncture analgesia [21]. A recent study found that electroacupuncture therapy can protect against cerebral ischemia by activating nAChR *α*7 in the brain, thereby suppressing neuroinflammation [22]. The findings in this study are very similar, noting that electroacupuncture stimulation resulted in increased levels of nAChR *α*7 in both the spinal cord and the PAG and decreased the levels of inflammatory factors (COX2 and Iba1). It can be speculated that the reduction of inflammatory response is the key mechanism of electroacupuncture analgesia.

The spinal cord and the midbrain PAG form part of the pain transmission and modulation center. The spinal cord sends messages from the body to the brain, while the PAG is responsible for part of the body's autonomic functions and pain control [23]. The nicotinic acetylcholine receptors nAChR *α*7, nAChR *α*4, and nAChR *β*2 are widely researched due to their potential as pain regulators and treatment points. nAChRs lead to antinociception in many central and peripheral areas. It is involved in the cholinergic anti-inflammatory pathway [24]. In one study, electroacupuncture significantly enhanced the nAChR *α*7 expression in the spinal dorsal horn after surgery, which resulted in an analgesic effect [25]. The results of this current study further confirmed that the analgesic effects of electroacupuncture might be achieved by increasing the expression of nAChR *α*7.

The nAChR *α*4 and nAChR *β*2 receptors are located throughout the central and peripheral nervous systems and take part in the descending inhibitory pain pathway in the central nervous system. nAChR *α*4 and nAChR *β*2 activation prompt the release of serotonin, norepinephrine, and GABA, all crucial neurotransmitters in the inhibitory pain pathway, expressed at both brain and spinal cord levels [26, 27]. Additionally, nAChR *α*4 and nAChR *β*2 may also play a role in chronic inflammatory pain due to their involvement in inflammatory cells [28]. In a prior study, electroacupuncture treatment significantly increased the expression of nAChR *β*2 protein [29], similar to what was demonstrated in this trial.

## 5. Limitation

Chronic pain inhibits glycine receptor *α*3 (GLRA3), which reduces the inhibition of nociceptive neurons and increases the transmission of nociceptive stimuli. In contrast, the expression of GLRA3 produces analgesia [30]. This study showed the numerical decrease of GLRA3 after nicotine withdrawal and the increase after electroacupuncture treatment. However, it did not reach significance. A larger sample size may be needed to clarify the effect of GLRA3 in nicotine withdrawal and electroacupuncture.

## 6. Conclusion

This study reveals that chronic nicotine exposure, followed by abrupt nicotine withdrawal can lead to hyperalgesia. Electroacupuncture (EA) has a significant analgesic effect on pain sensitivity in nicotine withdrawal hyperalgesia rats. The results of this study suggest that the mechanism of analgesic effect of EA treatment: (1) EA stimulation increased the expression of nicotinic acetylcholine receptor *α*7 (nAChR *α*7) in the spinal cord and PAG; (2) EA stimulation also increased the expression of nAChR *α*4 and nAChR *β*2 in the spinal cord; (3) EA stimulation decreased the expression of COX2 and Iba1 inflammatory factors in the spinal cord and PAG. Therefore, electroacupuncture may be an appropriate analgesic treatment for chronic pain induced by nicotine withdrawal.

## Data Availability

The research data associated with this research paper can be obtained from the corresponding author upon reasonable request.

## Abbreviations

ST36: Acupuncture point of “Zusanli”
LR3: Acupuncture point of “Taichong”
COX2: Cyclooxygenase 2
GLRA3: Glycine receptor *α*3
Iba1: The marker of microglia ionized calcium-binding adaptor molecule 1
nAChR *α*7: Nicotinic acetylcholine receptor *α*7
nAChR *α*4, nAChR *β*2: Nicotinic acetylcholine receptors *α*4 and *β*2
CTR: Control group
NIC: Nicotine group
NIC + *W*: Nicotine withdrawal group
EA: Electroacupuncture.
